# Towards case-based medical learning in radiological decision making using content-based image retrieval

**DOI:** 10.1186/1472-6947-11-68

**Published:** 2011-10-27

**Authors:** Petra Welter, Thomas M Deserno, Benedikt Fischer, Rolf W Günther, Cord Spreckelsen

**Affiliations:** 1 Department of Medical Informatics, RWTH Aachen University of Technology, Aachen, Germany; 2 Department of Diagnostic Radiology, RWTH Aachen University Hospital, Aachen, Germany

## Abstract

**Background:**

Radiologists' training is based on intensive practice and can be improved with the use of diagnostic training systems. However, existing systems typically require laboriously prepared training cases and lack integration into the clinical environment with a proper learning scenario. Consequently, diagnostic training systems advancing decision-making skills are not well established in radiological education.

**Methods:**

We investigated didactic concepts and appraised methods appropriate to the radiology domain, as follows: (i) Adult learning theories stress the importance of work-related practice gained in a team of problem-solvers; (ii) Case-based reasoning (CBR) parallels the human problem-solving process; (iii) Content-based image retrieval (CBIR) can be useful for computer-aided diagnosis (CAD). To overcome the known drawbacks of existing learning systems, we developed the concept of image-based case retrieval for radiological education (IBCR-RE). The IBCR-RE diagnostic training is embedded into a didactic framework based on the Seven Jump approach, which is well established in problem-based learning (PBL). In order to provide a learning environment that is as similar as possible to radiological practice, we have analysed the radiological workflow and environment.

**Results:**

We mapped the IBCR-RE diagnostic training approach into the Image Retrieval in Medical Applications (IRMA) framework, resulting in the proposed concept of the IRMAdiag training application. IRMAdiag makes use of the modular structure of IRMA and comprises (i) the IRMA core, i.e., the IRMA CBIR engine; and (ii) the IRMAcon viewer. We propose embedding IRMAdiag into hospital information technology (IT) infrastructure using the standard protocols Digital Imaging and Communications in Medicine (DICOM) and Health Level Seven (HL7). Furthermore, we present a case description and a scheme of planned evaluations to comprehensively assess the system.

**Conclusions:**

The IBCR-RE paradigm incorporates a novel combination of essential aspects of diagnostic learning in radiology: (i) Provision of work-relevant experiences in a training environment integrated into the radiologist's working context; (ii) Up-to-date training cases that do not require cumbersome preparation because they are provided by routinely generated electronic medical records; (iii) Support of the way adults learn while remaining suitable for the patient- and problem-oriented nature of medicine. Future work will address unanswered questions to complete the implementation of the IRMAdiag trainer.

## Background

The diagnostic process plays a predominant role in the everyday work of a radiologist. As is usual in medicine, this process requires not only explicit, formalized domain knowledge but also implicit or tacit knowledge gained by experience [1]. Radiologists acquire expertise through intensive, long-lasting practice and professional collaboration, which is time-consuming and laborious. Furthermore, real patients are involved in the learning process, which is carried out under time pressure. A diagnostic training system with settings similar to those found in the real world could ease and accelerate the acquisition of diagnostic proficiency.

Medical education, e.g., residency training, relies on practical experience within health care institutions such as hospitals [2]. The majority of problems physicians have to deal with directly concern the patient (clinical cases). Therefore, the presentation of clinical cases in medical education is essential [3]. Problem-based learning (PBL) is known to provide positive effects in medical education, especially pertaining to its social and cognitive aspects [4]. A clinical case is a stimulus for learning, fostering problem-specific knowledge acquisition and improving problem-solving skills [3].

These factors speak in favour of developing a computer-assisted diagnostic training within a defined educational framework using real clinical cases that offer valuable work-relevant experiences and insight into clinical processes. Computer-based training is particularly applicable in radiology because daily work is typically done on a computer and routinely created electronic patient records can provide direct input for training cases. Many approaches to computer-assisted teaching and lectures in medicine have been published [5-8]. However, existing systems are usually based on specially prepared cases. Although real patients' medical records are typically used, their preparation often requires excessive additional effort. These learning systems are usually implemented as stand-alone applications, i.e., they are not integrated into the medical information systems of a particular radiology department. Thus, the resulting insight into clinical practice is limited [9].

Clinical problem solving involves, in part, an implicit or explicit use of experiences with similar problems or past cases. Typically, a radiologist unknowingly compares a new case to previously solved cases, thereby applying his or her diagnostic experience. The case-based reasoning (CBR) paradigm developed in the context of artificial intelligence intends to simulate this type of problem-solving behaviour [10]. CBR in medicine is typically applied to clinical decision support [11], including diagnostic decision support systems. CBR comes with an apt explanation and justification of the solutions represented by the previous cases and their similarities to the current case. Therefore, it avoids the so-called "black box" solution, which may impede the physician's understanding.

The patient-oriented approach of CBR can be considered a complement to evidence-based medicine (EbM). EbM relies on the availability of clinical studies and supports the application of recent evidence (provided by meta-studies of randomized controlled trials or weaker forms of empirical support) into the clinical practice, while considering the individual patient's needs [12]. Clearly, clinical studies are limited to the investigation of distinct, well-defined alternatives. This limitation is prone to difficulties, due to inevitable uncertainties and vagueness in the medical realm [13]. Medical learning systems based on CBR teach the learners the importance of utilizing case-specific knowledge in combination with general textbook knowledge [14]. Although CBR has become a very active area of research in health science, CBR systems are rarely found in routine clinical use [13].

In addition to anamnesis, the initial point of a radiological diagnosis is the examination image created by an imaging modality for medical examination. Here, content-based image retrieval (CBIR) [15] is able to provide computer-assisted diagnostic support. CBIR offers access to image repositories by means of visual features derived from the image pixels. The potential benefits of CBIR for medical education have been confirmed [16-18]. The Casimage project features the creation of annotated teaching file case bases. It applies CBIR as a query extension to complement text-based searches [19]. The ASSERT system has been employed as a learning tool in radiology to teach different appearances of a particular lung disease using CBIR [17]. Seng and Mirisaee developed a CBR system for blood cell images selected by CBIR. They plan to use it for presenting pathology deviations in the undergraduate program of health sciences at the University of Malaya [18].

The aforementioned examples offer case repositories of pairs combining a given problem with its respective solution solely for presentation to the learners. ClinicalCases.org [20] includes a section of questions prepared by medical experts, e.g., to request the right diagnosis. The Open Distributed Internet Text Book (ODITEB) for tumour diagnosis [21] provides cases that are didactically prepared by means of expert-guided diagnostic tours.

The authoring of cases is a costly task, and case-based medical learning systems often provide only a limited number of cases due to the complexity and implementation effort [11]. Authoring environments exist for the creation of teaching files or case descriptions integrated into the picture archiving and communication systems (PACS), thus simplifying the selection and transfer of patient cases [19,22]. The approach of Abidi et al. aims to avoid the effort of authoring by automatically transforming electronic medical records (EMRs) into cases [23]. It assumes that EMRs are encoded in Extensible Markup Language (XML) format. Because many RIS/HIS records are based on HL7v2 and do not use the XML format for encoding EMRs, a format conversion will be necessary.

Different CBR learning scenarios have been investigated, such as e-learning systems for self-studies [20,21] and presentation systems guided by a teacher [17,18]. The McBAGEL (Multiple Case-Based Approach to Generative Environments for Learning) [24] is embedded into a variant of PBL and provides access to a case library for solving a presented problem. However, studies on McBAGEL showed that learners need more guidance than it provides because its educational concept does not include the tutor featured in PBL.

In the following study, we review theories of adult learning and education, case-based reasoning and problem-based learning, as well as content-based image retrieval in its application to diagnostic radiology training.

### Adult learning theories

The acquisition of expertise and the differences between novices and experts in medicine have been thoroughly studied. Experts commonly have more individual knowledge, whereas novices rather possess abstract and general knowledge [25]. Kushniruk et al. [26] observed that experts structure diagnostic knowledge into small sets of logically related disease schemas or "small worlds," allowing a rapidly generated, manageable number of tightly connected diagnostic hypotheses. Small worlds enable the expert to handle large amounts of information, rule out competing hypotheses and focus on a few distinguishable findings. Experts notice features and meaningful patterns of information of which novices are not aware [27]. Furthermore, experts are able to relate problems to previous ones and reuse solutions [28].

According to Patel et al. [29], experts narrow uncertainties, whereas novices generate more hypotheses than required, due to their domain knowledge being insufficient to discriminate between hypotheses. Novices were not able to constrain the number of their sets of diagnostic hypotheses. Their knowledge was organized as a flat structure, resulting in a considerable search effort. Training makes novices more aware of the information in and structure of the given data [30]. The development of expertise requires learning rule-based knowledge (e.g., through books and lectures) as well as experience-based knowledge (e.g., through practice with real patients) [29].

The andragogical model of adult learning theory [31] is well accepted. It is based on the following four aspects, which differ from pedagogical theory:

(i) *Direction*: Adults tend to be self-directed and are characterized by self-employed learning;

(ii) *Source*: Training needs to incorporate the comprehensive reservoir of experience, as this is the richest source of adults' learning;

(iii) *Motivation*: The adult learner's motivation depends on her needs and interests; she is generally motivated to learn due to internal or intrinsic factors as opposed to external or extrinsic forces;

(iv) *Orientation*: Adult learning is life-, task-, or problem-centred as contrasted to a subject-matter orientation; learning units are life situations, not objects.

In conclusion, an effective learning situation for adults should have core characteristics as follows: (i) enabling practice and experiences, (ii) a problem-centred approach, and (iii) immediate use resulting from the learning effort.

As a consequence, medical learning should offer work-related experiences with patient problems and a conceptual framework for understanding the varied experiences [11]. Problem-based training does not only support the way adults learn but also suits the nature of medicine, which is essentially patient- and problem-oriented.

### Problem-based learning (PBL)

PBL is a method of active learning based on the investigation and solution of real-world problems [3] and is often used in higher education [32]. Learners work in small collaborative groups as self-directed problem-solvers. Tutors guide the learning process and provide orientation [3]; they must carefully account for the prior knowledge and the actual working hypotheses of the learners [33]. Learners are not told how to proceed but have to self-decide which steps to take in solving a given problem.

PBL supports increased motivation [3], critical thinking and creative skills. Empirical studies of PBL have demonstrated that PBL fosters learners' capability of applying their knowledge to novel problems [34]. The collective sharing of experience postulated by PBL positively influences decision making [25]. As a consequence, PBL bridges the gap between theoretic knowledge and professional competencies [35,36].

Since its beginnings in 1969 at McMaster University, Canada, PBL has been applied to the curricula of many medical schools [3,37]. In 1974, PBL was introduced at the medical school of Maastricht University, where the Seven Jump procedure was developed. After presenting a problem to learners, it comprises the following steps [38]:

#### First meeting of the learners' group together with their tutor

1. **Clarify problem description**: Explain unknown terms and concepts.

2. **Define problem**: List the phenomena to be explained.

3. **Analyse problem**: Brainstorm different explanations of the phenomena using prior knowledge and common sense.

4. **Construct a working hypothesis**: Criticize the proposed explanations and produce a coherent description of the processes that probably underlie the phenomena.

5. **Formulate learning objectives**: Define goals of self-directed learning and delegate tasks to each learner in the group.

#### Individual work outside of the group

6. **Self-directed study**: Finish your job and fill the gaps in your knowledge through self-study (approximately two days).

#### Second meeting of the group together with their tutor

7. **Report, evaluate and synthesize**: Share findings with your group, discuss the results and try to synthesize the knowledge acquired into a comprehensive explanation for the phenomena.

Real-world situations, especially in the medical field, may lead to ill-defined problems (e.g., inaccurate descriptions of patients) or incompletely understood problems (e.g., missing explanations of underlying mechanisms). These situations may cause confusion and irritation to learners trying to solve the problems by exclusively using rule- or literature-based knowledge. In this case, learning from already-experienced cases may provide help and additional insight. CBR adopts this approach. Furthermore, cases elaborated in PBL may be used for reasoning and solving further cases in the future, thus promoting CBR [39,40].

### Case-based reasoning (CBR)

CBR is a problem solving and experience-based reasoning paradigm based on an analogy to the human capability to solve new problems by learning from past experience [10]. In CBR terminology, a case usually denotes a pair: a problem situation and its corresponding solution. The four main steps of the CBR process are: retrieve - reuse - revise - retain, as described in the CBR cycle [10] (Table 1).

**Table 1:** Principal steps of the CBR cycle [10]

Step	Description
Retrieve	Suitable reference cases are identified in a known set of cases based on their similarity to the original case.

Reuse	Knowledge and information of the retrieved cases is used toward the solution of the original case. The solution of the retrieved case may have to be adapted to the original case prior to using it. The result of the reuse process is a suggested solution.

Revise	The solution is tested, e.g., applied to the real world or evaluated by a teacher. If needed, the solution is adjusted. The result of the revision process is a confirmed solution.

Retain	The new experience is incorporated into the system for future retrieval by storing the newly learned case in the case database or modifying an existing case.

A domain might be suitable for CBR application if records of previously solved problems exist that are viewed as an asset, and if experience is at least as valuable as textbook knowledge [41]. Medical domains are extremely difficult to model through logical formalization [42] because medicine is a descriptive and experimental science for which complete causal models are not available [13]. Therefore, an established strategy of medical problem solving is to use past cases as prototypical patterns. Solved cases represent knowledge and experts' clinical experiences that provide starting points for new problems. The retrieval of relevant cases is directly linked to the data representation of cases and the applied similarity measure that evaluates the usefulness of cases regarding the current problem.

### Content-based image retrieval (CBIR)

The predominant role of images, especially in radiology, has led to an increasing interest in the use of CBIR techniques in medical applications [16,43]. CBIR identifies images by their content, making use of visual information for retrieval [15,43]. In particular, features such as colour, shape and texture are used to index images. A query by example (QBE) aims to retrieve relevant images starting with a given image by comparing the features of the query image with the images in the database.

According to Eakins and Graham, image retrieval can be classified in three levels of increasing complexity [43]:

#### Level 1 - retrieval by primitive features

Features from the first level include, e.g., colour, texture, shape and the spatial location of objects. These low-level features are automatically extracted from the image without any further information.

#### Level 2 - retrieval by logical or derived features

This level focuses on the identity of image objects. A search is conducted for either an object of a particular type ("images containing a kidney") or an individual object with a given name ("images of patient Mr. X"). Additional knowledge is necessary to learn, for example, that a certain structure is a kidney.

#### Level 3 - retrieval of abstract attributes

This level assumes high-level reasoning about the meaning and purpose of objects or scenes. It includes retrieval of images, e.g., of events ("images of childbirth"), types of activity ("images of an ultrasound examination") or expressing emotions or abstract terms (e.g., joy or fear).

Depending on the image retrieval level, general background or domain knowledge is applied, e.g., general knowledge of the appearance of a kidney. Benchmarking of content-based image classification systems focusing on medical images has been tracked at the Image Cross-Language Evaluation Forum (ImageCLEF) [44]. The "bag-of-visual words" approach recently demonstrated a very good performance in CBIR [45,46]. This Level 1 method detects salient image regions, extracts their features and clusters these features into so-called "visual words" to be used for image description. In the context of computer-aided diagnosis (CAD), a typical application of CBIR supports differential diagnosis, i.e., distinguishing between two or more diseases through systematic comparison.

### Objectives

Although learning from past cases for future treatments has become increasingly interesting because an increasing number of cases are available in electronic format [47], a meta-concept of such systems has not yet been developed. Based on both medical education and image processing expertise, this paper presents the concept of a diagnostic training for novice physicians, e.g., residents of radiology. Our approach suggests a diagnostic training system adapted to the real-world context of radiologists with respect to the patient cases used, the radiological software systems and the clinical workflow. It is not used for diagnostic guidance in the radiological routine. We propose applying CBIR to identify known patient cases containing an image similar to the current one, where similarity is defined by characteristics of the image content. In the future, this step will be replaced by combined visual and textual retrieval, which will further improve query completion and system quality in general. Our approach establishes a comprehensive conception of integrated CBIR and CBR paradigms, referred to as image-based case retrieval for radiological education (IBCR-RE). IBCR-RE is designed for full integration into the PACS and hospital/radiological information systems (RIS/HIS). Furthermore, the diagnostic training is embedded into a sound learning scenario based on Seven Jump and PBL.

## Methods

The purpose of the presented approach is to offer diagnostic training to radiology novices, e.g., radiology residents. Considering results of adult learning techniques and the deficiencies of the investigated learning systems, we identified the following requirements for the target radiological training system:

(i) *Environment*: The learning environment must be integrated into the radiological routine, offering realistic experiences;

(ii) *Scenario*: The learning scenario must respect the characteristics of adult learning and offer a clear educational procedure;

(iii) *Cases*: The construction of training cases must require minimum effort and the retrieval of similar cases must adopt typical techniques used by radiologists;

(iv) *Design*: System design must be general and applicable to arbitrary clinical environments and CBIR systems.

In the following sections we investigate solutions to fulfil these requirements. We describe our considerations regarding the learning environment and show the adapted Seven Jump learning scenario. Furthermore, our concept of integrating CBIR with CBR and the way it considers acquisition and retrieval of cases is described. Finally, the system design is illustrated.

### Clinical learning environment

The training system should preserve the clinical workflow as much as possible in order to provide real-world scenarios and work-relevant experiences. Radiologists' clinical workspace is determined by the use of a PACS as part of the RIS. A PACS comprises an image management and communication system for image acquisition, archiving, communication, retrieval, processing, distribution and display. It manages images produced by imaging modalities and related patient data from RIS or HIS, depending on the individual clinical setting [48]. In the following text, the notation RIS/HIS will be used when a clear distinction between the two is not relevant to the context of radiological education. Diagnostic findings from different clinical departments, e.g., pathology, laboratory or endoscopy, are usually managed by RIS/HIS, e.g., using a central repository.

**Figure 1:**
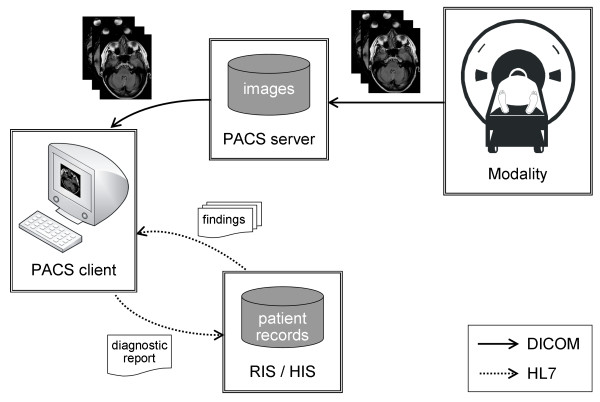
**Principal overview of the radiological workflow of creating a diagnosis**. The diagnostic findings are based on (i) examination images acquired by imaging modalities and retrieved from the PACS, and (ii) diagnostic findings from patient records retrieved from RIS/HIS. Arrows depict the data flow.

The typical radiology workstation offers access to the PACS, RIS and HIS [49,50]. The radiologist performs an image reading and prepares a description of his findings using a PACS client, retrieves further diagnostic findings from other departments and laboratories utilizing a RIS/HIS client and reports his diagnostic findings through a RIS/HIS interface (Figure 1). The network protocols used are DICOM to enable communication between the PACS and Health Level Seven (HL7) [51] and the RIS and HIS.

**Figure 2:**
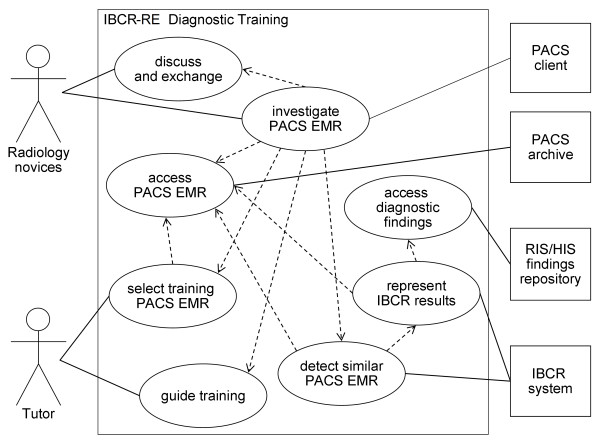
**UML use case diagram of IBCR-RE diagnostic training**. The diagram illustrates interaction and functionality of the involved components, namely the radiology novices and tutor on one side and the clinical systems and IBCR-RE system on the other. Lines denote actor-associations; dashed lines include relationships.

In the clinical environment depicted, there are several roles and systems involved in the diagnostic training process. Their responsibilities, requirements and relationships are described as follows (Figure 2):

• **PACS/RIS/HIS**: The PACS, RIS and HIS are used routinely by radiologists. The examination images and relevant findings are archived using electronic medical records. During the IBCR-RE diagnostic training, the PACS/RIS/HIS supply access to EMRs.

• **IBCR-RE system**: A CBIR engine retrieves image files from the PACS that fulfil the engine's similarity criteria and presents them to the radiology novices. Therefore, the IBCR-RE system requires a DICOM interface. Diagnostic findings from RIS/HIS are retrieved on demand to provide further insights. Therefore, this process demands HL7 interfaces.

• **PACS client**: The PACS client running on the radiologist's diagnostic workstation is used to interconnect the system components. A plug-in offers the possibility to initiate the IBCR-RE for the detection of PACS patient cases similar to the current patient context.

• **Tutor**: The trainer is preferably a radiologist. The trainer selects suitable completed patients' cases from the PACS to be used as training cases. The cases should be consistent with the novices' knowledge level. During the training session, the trainer offers guidance, e.g., in assessing the relevance of the solution to the training case.

• **Group of novices**: A small group of less than ten radiology novices investigates the proposed training cases within the PACS client. During this process, the novices discuss diagnoses and exchange knowledge relevant to the cases.

DICOM provides a standard encoding of structured data by means of the Structured Reporting (SR) format [52,53]. DICOM SR is an approved standard for exchanging CAD results in the clinical context, e.g., for mammography [54,55], which can be stored to and retrieved from the PACS. Due to the various advantages of this approach [56,53], IHE employs DICOM SR in several integration profiles [57,58]. Supporting a convenient integration into clinical environments, CBIR results used in the suggested IBCR-RE diagnostic training are encoded and exchanged as DICOM SR documents.

### Adapted Seven Jump diagnostic learning scenario

The learning scenario is established as a modified Seven Jump process carried out by small groups guided by a tutor in a single meeting at a radiological workstation:

1. **Clarify problem description**: The tutor specifies the completed patient case providing the problem to be approached in the training. The radiology novices open the case in the PACS client. Novices unfamiliar with the PACS client are introduced to its handling. Unknown medical terms are explained.

2. **Define problem**: The radiology novices determine the relevant findings in the examination image that need to be explained. This step will require assistance from the tutor because the identification of findings is a challenging task, even for experienced radiologists.

3. **Analyse problem**: From their current knowledge, the novices brainstorm to identify possible explanations.

4. **Construct a working hypothesis**: Applying a critical view to the identified explanations, the novices make a coherent diagnosis.

5. **Formulate learning objectives**: The novices list all open questions regarding their diagnosis. These issues serve as a task list for the next step.

6. **Explore similar patient cases**: The novices retrieve similar patient cases and compare them with respect to their differing and shared characteristics. They also explore the diagnostic reports of the identified cases created in diverse clinical departments, which may provide information on different aspects of the case. This may lead to questions requiring further information on the original case that the tutor may answer using the corresponding diagnostic reports.

7. **Report, evaluate and synthesize**: Diagnoses are shared and discussed with other groups. The training ends with revised diagnostic findings affirmed by the tutor.

In step 6, the novices require access to similar patient cases in the PACS. This access can be provided by a CBIR engine, as described in the next section.

### Image-based case retrieval for radiological education (IBCR-RE)

As previously stated, existing systems simply extend CBR by CBIR as an alternative query method. In contrast, our approach, which integrates CBIR and CBR paradigms to address medical learning in radiology, refers to multiple aspects, including case representation and the CBR cycle.

Each medical examination is regarded individually. The corresponding patient case is characterized by features that are inevitably lost during the creation of a generalized case [25] that might result from case adaptation. The patient's medical record contains both the "situation" (observed symptoms and findings) and the "solution" provided by medical experts (diagnosis, prognosis, treatment plan) [23]. Therefore, a case in IBCR-RE is generated based on a patient case from the radiological practice. In this context, a "case" refers to a PACS DICOM study, usually created on the basis of a patient's new disease together with the available diagnostic reports from RIS/HIS corresponding to the DICOM study comprising the patient's complete history of clinical treatment. The use of medical patient records provides a realistic simulation because physicians treat individual patients individually.

The integration of CBIR into the CBR cycle requires the following adaptations:

• **Retrieve**: Each diagnosis typically starts with anamnesis and analysis of the examination image. The image as starting point supports the concept of indexing and retrieving a case by its image features. The index is defined by the type of the extracted image features.

CBIR aims to retrieve a set of cases that contain images similar to the one being diagnosed (see discussion on limitations). As decision making cannot be fully automated [59] and physicians wish to see and interpret all specific details themselves [14], the retrieval is not restricted to the most similar image but returns a set of similar images along with their similarity scores.

**Reuse**: In CBR, cases are either employed directly (without modification) or automatically adapted to the current case (i.e., transferred to the new situation, e.g., dosing of medication for a child is adjusted for an adult, applying certain rules) before they are presented to the user. Physicians usually prefer to reason about current patients themselves [14]. Particularly in medical decision support systems, it is common to find examples of CBR tools that leave the responsibility of providing an interpretation and of proposing a solution to the user [1]. In fact, the adaptation task is the main problem in CBR [14]. It is an even more pressing issue in medicine because all differences between the current and the corresponding similar cases must be considered and can be difficult to capture [14].

Leake suggested the alternative method of adapting the context instead of the case [60]. Explaining a case's relevance shows how the case applies to a certain problem without modifying it. When using IBCR-RE, medical cases containing similar images are presented directly. The radiology novice establishes an association between the suggested cases and the original case that may lead to an adapted context.

• **Revise**: This step in the CBR cycle is inherited by the IBCR-RE adaptation without modifications. The elaborated solution must be verified.

• **Retain**: The adaptation of the training case created by the radiology novices will not be stored back to the PACS for usage in following retrievals. Preserving the PACS data will ensure that the routine operations of the radiology department are not affected.

• **Refine**: During exploration of the retrieved cases, relevance feedback and query refinement offer possibilities to interactively rate the retrieval result and re-run the retrieval process. Relevance feedback is important for accuracy [61] and improves performance [16,62]. Interactive refinement also gives radiology novices the opportunity to investigate different directions of solutions.

IBCR-RE incorporates the retrieve, reuse and revise steps of the traditional CBR cycle. Because adapted cases are not stored, the retain phase of the traditional CBR cycle is left out. Nonetheless, the added refinement process again leads to a cycle: retrieve - reuse - revise - refine, which in turn is embedded into the diagnostic learning scenario.

### System design

**Figure 3:**
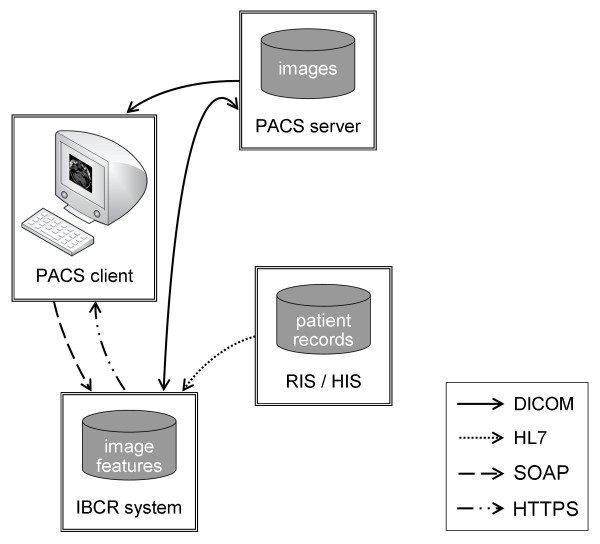
**System design of diagnostic training**. The design inserts the IBCR-RE system seamlessly into the standard radiological workflow (Figure 1). The IBCR-RE system is connected to the PACS client, the PACS server and RIS/HIS using standard protocols. Arrows depict data flow, starting from the source.

The diagnostic training system aims to embed the learning approach into the clinical context based on the following systems and communication paths (Figure 3):

(i) *PACS server*: Hosts the DICOM examination images that are transferred to the PACS client for viewing the initial training case and to the IBCR-RE system for retrieval of similar images. CBIR results encoded as DICOM SR documents are delivered to the IBCR-RE system for presentation purposes. Communication is established by DICOM.

(ii) *PACS client*: Triggers the IBCR-RE system using Simple Object Access Protocol (SOAP) messages containing the current patient context and listing the necessary DICOM information for retrieving the examination image from the PACS.

(iii) *RIS/HIS*: Hosts diagnostic findings that are transferred to the IBCR-RE system when referred by similar retrieved images. This delivers additional information on the case to be included in the presented CBIR results. HL7 messages are used for communication.

(iv) *IBCR-RE system*: Entails a CBIR engine for retrieval of patient cases containing an image rated similar to the query image and transfers the CBIR results encoded as DICOM SR documents to the PACS server for archival by DICOM commands. The prepared CBIR results containing corresponding diagnostic findings are transferred to the PACS client in order to present them to the radiology novice using the HTTPS protocol.

The use of standard protocols and the clearly established setting support the implementation of the diagnostic training in different clinical environments.

## Results

The IBCR-RE diagnostic learning scenario constituted by the adapted Seven Jump process provides a clear setting and procedure. In the following, partial implementation of the system and a walk-through of the concepts are presented in order to demonstrate the general feasibility of the proposed concept.

We use the Image Retrieval in Medical Applications (IRMA) framework [63] for CBIR. The IRMA framework offers a wide range of image features and retrieval methods, as well as their convenient extension. The resulting system is called "IRMAdiag trainer." In the following passages, we illustrate the IBCR-RE concept and its application using the IRMAdiag trainer. Then we describe two essential aspects of integration into the radiological environment, the CBIR DICOM SR template and the HL7 query for diagnostic findings. Finally, the IRMAcon viewer is presented.

### IRMAdiag trainer

**Figure 4:**
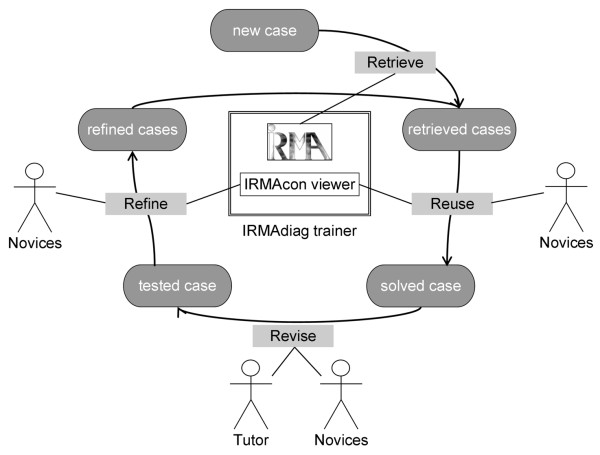
**Adapted case-based reasoning (CBR) cycle using the IRMAdiag trainer**. Application of image-based case retrieval for radiological education (IBCR-RE) in the adapted case-based reasoning (CBR) cycle for diagnostic training using the IRMAdiag trainer (IRMA core and IRMAcon viewer); Arrows depict process flow, lines illustrate roles and components involved in a step.

Our diagnostic learning system consists of two components: (i) the IRMA core [64] responsible for case indexing and retrieval; and (ii) the IRMAcon viewer [65], a combined DICOM and HL7 viewer that connects DICOM SR documents containing CBIR results with referenced diagnostic findings from RIS/HIS. The IRMAdiag trainer assists the radiology novices in step 6 of the adapted Seven Jump process, i.e., "Explore similar patient cases." The IBCR-RE paradigm has been implemented using the IRMAdiag trainer as follows (Figure 4):

• **Retrieve**: The IRMAdiag trainer, i.e., IRMA core, processes the image included in a new patient case and retrieves patient cases from the PACS containing examination images that the IRMA core rates to be similar. The retrieval uses image features that have been extracted from examination images included in patient cases residing at the PACS. Because image features are usually processed by CBIR algorithms and have no relevance to the clinical operation of the PACS, they are stored in a separate database exclusively used by IRMA. In the IRMAdiag trainer, similarity is based on visual features and does not consider textual information.

• **Reuse**: The IRMAcon viewer presents the retrieved cases and similarity scores, together with the corresponding diagnostic findings, to the radiology novices. The novices select the aspects that they consider relevant to the solution of the new training case, resulting in a solved case.

• **Revise**: The novices test and evaluate their solution, and the tutor verifies correctness and validity. The result of this step is a tested case.

• **Refine**: The IRMA web interfaces include tools for relevance feedback [61] and may be applied to the IRMAcon viewer to offer sliders for modifying the similarity scores determined by the IRMA core. The relevance feedback presented as refined cases is passed on to the IRMA core for a re-run of the retrieval process.

### DICOM SR template for CBIR

The IRMAdiag trainer encodes CBIR results as DICOM SR documents. To simplify the processing of DICOM SR documents and avoid different encoding of the same content, DICOM SR templates [66] define valid items and value types. These templates facilitate the automated processing and interpretation of SR documents [67]. However, the CAD application of CBIR is not covered by the DICOM standard templates. An SR template meeting the special requirements of CBIR has been developed in previous work [68]. The template comprises the query image, the identified similar images along with their similarity scores and a description of the applied CBIR system. The resulting SR document containing CBIR results may be stored back into the PACS, provided that DICOM SR is included in the PACS' DICOM conformance statement. Alternatively, a dedicated DICOM application storing SR documents can be used.

### HL7 query for observation results

Communication with RIS/HIS is accomplished by HL7 messages. In the clinical context RIS/HIS usually manages message exchange with a dedicated communication server. That implies that all HL7 messages are sent to the communication server, e.g., specified by an IP address and a port. The header of the HL7 message contains the final destination specified as "Receiving Application," and the communication server forwards the message to its addressee.

HL7 messages are divided into mandatory and optional segments and fields, depending on the particular message type. Each message begins with a header segment introduced by the keyword "MSH." An HL7 query message is composed of a message header ("MSH") followed by a query definition ("QRD") and a query filter ("QRF"). Message type QRYˆR02, "Query For Results Of Observation", requests diagnostic findings that are returned by message type ORFˆR04. The diagnostic information is then extracted from the received message and displayed to the radiologist. Reports included in an ORFˆR04 message may be plain text or binary format, e.g., Portable Document Format (PDF) or Microsoft Word, usually encoded as Base64 [69] before transmission.

### IRMAcon viewer implementation

The IRMAcon viewer uses IRMA modules for a web-based graphical user interface (GUI) [68] that has been adapted for the representation of CBIR results based on our CBIR SR template. IRMAcon comprises three components: (i) a PHP/DICOM web server application that interprets the CBIR SR document, determines examination information, collects referenced images, and calls the HL7 program; (ii) an HL7 program that retrieves the diagnostic findings and makes them available in their original formats; and (iii) a web browser on the client workstation that presents the resulting output.

IRMAcon uses the implementation of the DICOM protocol provided by the OFFIS DICOM-Toolkit (DCMTK) [70]. HL7 messages are implemented using HAPI (HL7 Application Programming Interface) [71]. SR documents are converted to XML by DCMTK and then parsed by the XML extension of PHP. To receive an image format suitable for representation in a common web browser, the retrieved DICOM images are converted to Portable Network Graphics (PNG) using the DCMTK library in the default setting.

We created a prototypical setting for our viewer that reduces the PACS and RIS/HIS to the relevant functionalities needed in our scenario. It includes a DICOM database provided by DCMTK configured with a DICOM Application Entity Title (AET) for accessing DICOM images and SR documents. The RIS and HIS are simulated by a simple HL7 application. IRMA has a SOAP interface for receiving requests and input data and returning results.

### IRMAcon viewer layout

**Figure 5:**
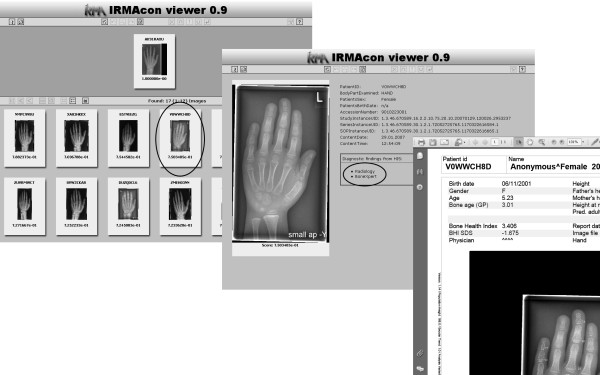
**Example output of the IRMAcon viewer**. The top window (a) shows an overview of CBIR results, and the middle window (b) presents details on a selected patient case; at the lower right corner (c) an associated document containing diagnostic findings selected from the list is presented.

Following Dayhoff et al., the principal goals of the clinicians' workstation design [72] are: (i) easy and quick comprehension supported by structuring and limitation to only relevant data; (ii) intuitive and simple handling supported by a simple navigation; and (iii) presentation adapted to the particular application, namely, aligned with the hanging of radiographs. The layout of the IRMAcon viewer (Figure 5) is aligned with these goals as follows.

The main window (Figure 5-a) predominantly presents images instead of textual data, which simplifies information capture. In the top area, the training examination image for diagnosis is shown. Similar images identified by the CBIR system are listed below in descending order of their similarity for easy comparison. This composition structures the information according to the identified relevance regarding the query image and is aligned with a physician's typical workflow. Navigation is enabled with arrows pointing to the left for switching to images more similar or to the right to images less similar.

Each downscaled image can be enlarged by a double click, which also provides detail (Figure 5-b), such as the examined body part, date of birth and sex. Corresponding diagnostic findings from the RIS/HIS are supplied after activating the respective button. Figure 5-c shows diagnostic findings created by the bone-age assessment software BoneXpert [73]. Providing details only on demand allows a compact overview, which further supports efficient capturing.

An example training session using the adapted Seven Jump scenario has been drafted to provide a summary. During preparation, the tutor selects a patient case that results in the IRMAdiag trainer in PACS cases containing images of sufficient similarity to the training task. We will assume the case of a girl born in 2003 who presents a growth disturbance resulting in a size that is much smaller than the average. The diagnosis will propose the type and cause of the disorder and suggest probable growth development. The determination of skeletal maturity, also referred to as bone age assessment (BAA), allows us to estimate children's heights in adulthood and to diagnose and track endocrine disorders or paediatric syndromes, usually based on hand radiographs [73]. The selected case contains an x-ray imaging of the left hand in anterioposterior orientation.

1. **Clarify problem description**: The tutor names the selected patient case by giving first and last name, date of birth and the unique DICOM Study ID. The tutor explains the problem and the requested generation of a bone age assessment. The radiology novices search for the patient case in the PACS client.

2. **Define problem**: The radiology novices analyse typical distinguishing marks in the given hand radiograph, e.g., the epiphyseal regions, and the tutor assists in the proper evaluation.

3. **Analyse problem**: During a brainstorming session, the novices collect possible solutions based on their present knowledge.

4. **Construct a working hypothesis**: Based on their analysis, the novices decide on a certain bone age.

5. **Formulate learning objectives**: The novices will probably encounter uncertainties regarding the correctness of their hypothesis and specify questions accordingly.

6. **Explore similar patient cases**: The novices start the IRMAdiag trainer by selecting the corresponding command from the context menu of the current patient case in the PACS client. This command creates a SOAP message containing the DICOM UID of the case immediately sent to the IRMAdiag trainer. The IRMAdiag trainer retrieves the corresponding image from the PACS, converts it to the PNG format and extracts visual features, which are then compared to the features of already processed PACS cases contained in the IRMAdiag database. In the following, the resulting similar images and their corresponding PACS patient cases and diagnostic reports from RIS/HIS are listed in the IRMAdiag viewer. Figure 5-b shows an exemplary output with diagnostic findings retrieved from HIS of different origins: (i) BoneXpert, containing the computed BAA results by the commercial software (Figure 5-c), and (ii) radiology, presenting the diagnosis provided by the responsible radiologist. The novices may elaborate answers to their questions in step 5. The tutor offers assistance if the novices have problems concerning the examined patient cases.

7. **Report, evaluate and synthesize**: Each group presents its results, subsequently discussed with the other participating groups. The tutor finally comments on the bone age assessment and provides relevant explanations.

## Discussion

The special characteristics of radiological practice, particularly the daily use of the computer in the radiological routine, provide an ideal application context for the proposed diagnostic training system. The importance of images fosters the application of CBIR methods for the retrieval of similar patient cases. The strong correlation between the quality of diagnoses and the degree of experience provides a sound motivation for the use of experience-based knowledge as provided by CBR.

However, the performance of current CBIR systems is insufficient for a general application in CAD in general, as affirmed by the poor results, especially on the diagnosis level, achieved at ImageCLEF [74]. Furthermore, CBIR systems are typically specialized, e.g., in a certain anatomic region or modality. This limitation impedes its general application in radiology and argues for a dedicated use depending on the capabilities of the particular CBIR system. IRMA, for example, focuses on hand radiographs. Furthermore, IRMA does not provide adapted algorithms for integrated processing of tomographic images. Depeursinge et al. developed a multimodal distance measure for lung tomography images using automatically extracted three-dimensional regions [75]. The use of a combined similarity value for the images contained in a case is expected to further improve case retrieval. Approaches exist that, for example, determine a fusion of the single image-based similarity values of a case to calculate a combined case-based similarity value [76]. In order to cope with the various aspects of case similarity, according algorithms have to be investigated and added to IRMA using its convenient interface for extensions. To cope with the insufficient performance of CBIR systems in diagnostic training, the tutor may also use IBCR-RE to select the initial clinical case during preparation. If the set of cases similar to the original case retrieved by the system does not provide a reliable basis for the diagnostic training, another case will have to be chosen. Nonetheless, the IRMAdiag trainer addresses serious drawbacks of typical training systems regarding training cases, integration into radiologists' daily routine and learning scenarios. The proposed training does not claim to provide general diagnostic support.

Today's CBIR algorithms are unable to handle complete PACS databases that consist of several terabytes of data per year. A feature comparison for all images of a PACS database within a period of time acceptable to clinical routine is a problem that has not yet been solved in a satisfying way. As an intermediate step, the IRMAdiag trainer can be extended with a learning module that supports the tutor in selecting a representative set of patient cases to be used as CBIR reference database instead of automatically inserting each new PACS patient case into IRMAdiag.

Due to the use of patient cases from the PACS, the IRMAdiag trainer has to take the security and privacy of patient data into account, and regulatory affairs differ internationally. Many European countries prohibit the use of patients' data for educational purposes unless an ethics committee agrees on the use. Other countries may allow, for example, radiology residents to access patients' EMRs in the context of their training, also when retrieved by a computer training system. As a consequence, specific legal regulations must be considered very carefully. Otherwise, use of the IRMAdiag trainer will not be allowed. The @neurIST project proposes methods such as de-identification, data minimization, aggregation and pseudonymisation to achieve the required anonymity of patient data [77]. Patient data used in the IRMAdiag trainer is retrieved from the PACS and RIS/HIS and the effort required for the conversion of free text into anonymous form after retrieval and before representation to the learner has to be investigated. Anonymisation modules for DICOM data are available and will be adopted from the @neurIST project or from open source libraries, e.g., the Medical Imaging Resource Center (MIRC) DICOM anonymiser http://mirc.rsna.org.

The education theories analysed in the course of this project indicate that the IRMAdiag trainer (i) provides a suitable means of supporting the learning process of radiology novices as described in the andragogical model, (ii) increases motivation and (iii) supports the acquisition of professional competencies. The IBCR-RE training fosters the application of theoretical knowledge gained during the learners' studies. The IRMAdiag trainer does not provide prepared cases that are appropriately annotated for learners starting their studies. Patient cases taken from the PACS may present complexities or unrelated problems that can distract or confuse the radiology novice [3]. Medical learners are usually not familiar with judging the relevance of suggested solutions [11]. As a consequence, the proposed diagnostic training should be scheduled when radiological knowledge has already been acquired, e.g., during a radiology residency program.

Radiology typically suffers from limited follow-up of diagnoses because patient treatment is usually carried out by other physicians. The IRMAdiag trainer offers access to diagnostic reports from different clinical departments, thereby providing different aspects of a patient's (clinical) treatment but not necessarily composing a complete picture. Furthermore, data, diagnoses and treatment plans from real patient cases are not certain to be complete and error-free, and treatments described in the retrieved cases may be questionable or even harmful. If the learners miss these ambiguities, the tutor must indicate and address them to prevent the learners from repeating mistakes. The learners have to develop the means of intensive inspection and scrutiny that they will also need as qualified doctors. Furthermore, they will learn the importance of data quality issues early [78]. To achieve these goals, a tutor is indispensable to appropriately support the learners. This need puts the tutor in a very important position because the quality of the training depends on the tutor's knowledge and experience. Mistakes in a retrieved case may not be discovered by learners or tutor, potentially leading to learners repeating those mistakes in the future. Even a very experienced radiologist cannot guarantee that all abnormalities will be discovered in medical images, particularly in the more complex cross-sectional data sets. Thus, the use of patient data from the PACS bears the problem of potentially vague datasets without definite and clear-cut diagnoses, and learners should not fully rely on the retrieved diagnoses.

**Figure 6:**
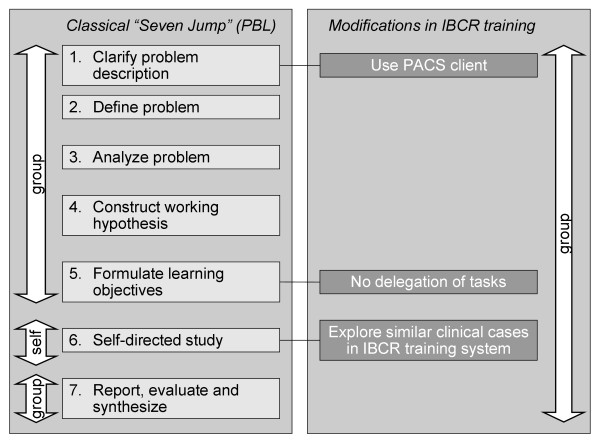
**Comparison of the "Seven Jump" and IBCR-RE training**. Differences between the classical "Seven Jump" from PBL and the modifications made for the diagnostic training.

The Seven Jump process of PBL had to be modified in order to provide a learning scenario adapted to the diagnostic training in the following way (Figure 6):

(i) *Use of tools*: The Seven Jump process does not rely on the use of computer systems, though it does not prohibit it. In step 1, the IBCR-RE training establishes a realistic working environment and uses the PACS client to present the initial problem. In step 6, the IBCR-RE system delivers realistic patient cases as a basis for the novices' diagnosis.

(ii) *Learning context*: The classical PBL scenario is divided into three settings. In the first meeting with the tutor, steps 1 through 5 are carried out. Tasks are formulated and assigned to the single learners in step 5 for self-directed study in step 6. Step 6 has to be performed individually and may take about two days of further study. In the second meeting with the tutor, step 7 is carried out. In contrast, the IBCR-RE training is performed at one single meeting. The gathered questions are not assigned to the learners in step 5, but they are examined together in step 6. Due to the special situation in the radiological domain in which the identification of findings and the appraisal of diagnostic reports requires experienced proficiency, the tutor offers appropriate assistance to the novices during the steps "Clarify problem description," "Define problem," and "Report, evaluate and synthesize."

In summary, the aspects by which the design of the suggested learning system fulfils the requirements identified for a radiological training approach are given in the following:

(i) *Environment*: In order to enable a real-world scenario, the system needs to be embedded into the clinical practice of a radiologist, i.e., the PACS environment. Our learning system not only provides a collection of pathologies for investigation and comparison but also focuses on the actual diagnostic training. This encompasses the steps of making a report on diagnostic findings included in the radiological process [79]: perception and interpretation of the relevant findings;

(ii) *Scenario*: The learning system is embedded into a sound educational frame based on the Seven Jump process of PBL. Work-related experiences are enabled using real patient problems, and experience-based knowledge is supported by CBR;

(iii) *Cases*: Teaching cases are automatically acquired from patient cases stored in the PACS. Suitable reference cases are identified by the content of the examination images using CBIR methods;

(iv) *Implementation*: The system architecture is based on clinical standards and contains exchangeable components. This supports its general application to various clinical environments and CBIR systems.

Several studies have shown that combining CBIR with natural language processing (NLP) of medical unstructured text (e.g., anamnesis, diagnosis) associated with the images and hosted in the EMR may significantly improve query completion [80,81]. Case retrieval based on both image and contextual information has been used, e.g., by Quellec et al., who developed a framework for the retrieval of cases in medical databases [82]. Results from ImageCLEF show that combining textual and visual information is important for effective retrieval [74]. However, this is not seen as a contraindication but as a future enrichment to the proposed system, as the CBIR frameworks such as IRMA are capable of including NLP principles as well [83].

In the following section, we present the planned evaluation scheme of the IRMAdiag trainer. We suggest two tutors (radiologists with at least 2-3 years of experience) with two training groups each. Their aims are as follows:

(i) Appraise the learning outcome in terms of self-ascribed progress and confidence in their own diagnostic skills as viewed by the learners;

(ii) Check whether tutors consider preparation for the training acceptable with respect to time and effort and whether, in their opinions, the IRMAdiag trainer provides a supportive setting for their training of novice radiologists;

(iii) Determine the proper size of a training group;

(iv) Measure the time used by the tutor and the novices at each step of the adapted Seven Jump approach.

A pilot study will be carried out to (a) fix obvious problems with the implementation of the scenario, and (b) to unveil critical aspects to then be addressed in the main study. During the pilot study, a group of radiology trainers acting as learners will perform a cognitive walkthrough, including the selection of training cases supported by the IRMAdiag trainer.

We further plan to examine how much the IRMAdiag trainer helps improve learners' diagnostic capabilities. An exam (containing, for example, the diagnosis of selected patient cases) will be performed comparing two groups of learning radiologists, of which only one will use the IRMAdiag trainer.

The evaluation will be framed by questionnaires at the beginning and end of the Seven Jump process. The members of the training groups will assess their attitude toward the learning approach and their self-ascribed levels of expertise with Likert scale items presented to them identically before and after the training. The tutors answer a questionnaire containing both Likert scale and free-text items after the training. We further expect valuable insights from a focus group of radiology trainers addressing the strength and weaknesses of the approach after implementing and using the training. The qualitative data (protocol of the focus group and free-text answers) will be assessed applying appropriate methods of qualitative research [84].

## Conclusions

The presented diagnostic learning system for radiology novices compensates for the drawbacks of existing systems as follows: (i) Real patient cases and integration into the clinical environment allow a real-world setting providing realistic experiences; (ii) By using patient cases from the PACS, bottlenecks in case preparation are reduced because new cases are stored automatically during the everyday working process; (iii) The diagnostic training is embedded into a sound learning scenario that is similar to the approved Seven Jump process from PBL.

PBL enables learners and novice physicians to acquire both scientific knowledge and professional skills, including lifelong learning, teamwork and social responsibility [85]. CBR simulates the human problem-solving strategy of adapting known experiences or solutions to diagnostic decision support systems. Our diagnostic learning system takes full advantage of PBL and CBR. In order to follow the usual approach of a radiologist who compares images from past patient cases with the current problem to assess the corresponding diagnoses, we developed IBCR-RE, the multi-level integration of CBIR with CBR. The retrieval of similar patient cases based on image content profits from the predominant role of images in the radiology domain. The novel IBCR-RE diagnostic training does not only apply CBIR to find potentially relevant cases for comparison, but combines CBIR with the paradigm of CBR.

In a feasibility study of our integrative learning system, we partially implemented the IRMAdiag trainer. The IRMAdiag trainer not only provides a collection of pathologies but also simulates the radiological workflow. The use of EMRs from the PACS as teaching cases, the integration of the PACS client, the application of standard clinical protocols for communicating and formatting results, and the retrieval of patient data and diagnostic findings from RIS/HIS establish the real-world setting of a radiologist and provide realistic work-related experiences. The use of approved methods and clinical standards establishes a solid and reliable basis for the IRMAdiag trainer. In the future, we plan to validate the IRMAdiag's quality through experiments with inexperienced, novice radiology physicians.

We presented a novel modification of the well-established Seven Jump approach of PBL for use in radiological training. Embedded into a proper learning scenario, partly implemented and integrated into the clinical context, the IRMAdiag trainer could show how CBIR can benefit radiological training. The IRMAdiag trainer is based on approved learning methods applied in both protected and realistic contexts and represents a modern training concept to enrich the range of current medical training.

## List of abbreviations and acronyms

AET: Application Entity Title (DICOM); CAD: Computer-aided diagnosis; CBIR: Content-based image retrieval; CBR: Case-based reasoning; DCMTK: DICOM-Toolkit; DICOM: Digital Imaging and Communications in Medicine; EbM: Evidence-based medicine; EMR: Electronic medical record; HIS: Hospital information system; HL7: Health Level 7; IBCR-RE: Image-based case retrieval for radiological education; IHE: Integrating the Healthcare Enterprise; IRMA: Image Retrieval in Medical Applications; PACS: Picture archiving and communication system; PBL: Problem-based learning; PNG: Portable Network Graphics; QBE: Query by example; RIS: Radiology information system; SOAP: Simple Object Access Protocol; SR: Structured Reporting (DICOM); XML: Extended Markup Language.

## Competing interests

The authors declare that they have no competing interests.

## Authors' contributions

PW developed the core of the presented concept comprising different aspects to which the co-authors contributed in the following way: (i) CS refined the education concept and it's embedding into a proper learning scenario; (ii) BF and TMD contributed to applying the concept to IRMA; (iii) RWG assessed the training's feasibility in radiology departments. The denotation "IBCR-RE" was created by TMD and PW. PW prepared the first draft of the article, which has been revised by CS, BF and TMD. All authors have read and approved the final manuscript.

## Pre-publication history

The pre-publication history for this paper can be accessed here:

http://www.biomedcentral.com/1472-6947/11/68/prepub
